# The impact of changing exposure to PM_2.5_ on mortality for US diplomats with multiple international relocations: a modelling study

**DOI:** 10.1186/s12940-024-01127-6

**Published:** 2024-10-22

**Authors:** Leslie Edwards, James Milner, Paul Wilkinson, Ai Milojevic

**Affiliations:** London School of Hygiene and Tropical Medicine, London, England, UK

**Keywords:** Mortality, Death, Relocation, Movers, Diplomats, Model, Cessation lag, Particulate matter, PM_2.5_, Mitigation

## Abstract

**Background:**

Current evidence linking long-term exposure to fine particulate matter (PM_2.5_) exposure and mortality is primarily based on persons that live in the same residence, city and/or country throughout the study, with few residential moves or relocations. We propose a novel method to quantify the health impacts of PM_2.5_ for United States (US) diplomats who regularly relocate to international cities with different PM_2.5_ levels.

**Methods:**

Life table methods were applied at an individual-level to US mortality statistics using the World Health Organization’s database of city-specific PM_2.5_ annual mean concentrations. Global Burden of Disease concentration-response (C-R) functions were used to estimate cause-specific mortality and days of life lost (DLL) for a range of illustrative 20-year diplomatic assignments for three age groups. Time lags between exposure and exposure-related mortality risks were applied. Sensitivity analysis of baseline mortality, exposure level, C-R functions and lags was conducted. The effect of mitigation measures, including the addition of air purifiers, was examined.

**Results:**

DLL due to PM_2.5_ exposure for a standard 20-year assignment ranged from 0.3 days for diplomats’ children to 84.1 days for older diplomats. DLL decreased when assignments in high PM_2.5_ cities were followed by assignments in low PM_2.5_ cities: 162.5 DLL when spending 20 years in high PM_2.5_ cities compared to 62.6 DLL when spending one of every four years (5 years total) in a high PM_2.5_ city for older male diplomats. Use of air purifiers and improved home tightness in polluted cities may halve DLL due to PM_2.5_ exposure. The results were highly sensitive to lag assumptions: DLL increased by 68% without inception lags and decreased by 59% without cessation lags for older male diplomats.

**Conclusion:**

We developed a model to quantify health impacts of changing PM_2.5_ exposure for a population with frequent relocations. Our model suggests that alternating assignments in high and low PM_2.5_ cities may help reduce PM_2.5_-related mortality burdens. Adding exposure mitigation at home may help reduce PM_2.5_ related mortality. Further research on outcome-specific lag structures is needed to improve the model.

**Supplementary Information:**

The online version contains supplementary material available at 10.1186/s12940-024-01127-6.

## Background

Strong evidence has been established that an increase in exposure to fine particulate matter air pollution equal to or smaller than 2.5 microns (PM_2.5_) is associated with an increase in the risk of mortality [1, 2]. A comprehensive systematic review of cohort studies reported that a 10 µg/m^3^ increase in long-term PM_2.5_ exposure is associated with an 8% (95% CI: 6%, 9%) increase in natural-cause mortality and each cause of mortality evaluated in the review showed statistically significant associations with PM_2.5_ exposure: 11% (9%, 14%) increase for circulatory disease, 16% (10%, 21%) increase for cerebrovascular disease, 10% (3%, 18%) increase for respiratory disease and 12% (7%, 16%) increase for lung cancer per the same increment in PM_2.5_ [3]. Another extensive systematic review including more than 25 years of cohort studies reported a similar order of excess mortality risk due to PM_2.5_ exposure: 8% (95% CI: 6%, 11%) increase in all-cause mortality, and cause-specific mortality including 11% (8%, 14%) for cardiopulmonary disease and 13% (7%, 20%) for lung cancer, per 10 µg/m^3^ increase in PM_2.5_, respectively [2]. Interestingly, the meta-analysis estimated indicated robust PM_2.5_-mortality associations, but with heterogeneity in the magnitude of associations among geographic regions (North America, Europe, and Asia). The Health Effects Institute conducted a comprehensive systematic review of the associations between long-term exposure to traffic-related air pollution (TRAP) and a wider range of adverse health outcomes including birth and respiratory outcomes for children [4]. The findings have provided an overall high or moderate-to-high level of confidence in the association between long-term exposure to TRAP and adverse health outcomes including all-cause mortality, mortality due to circulatory disease, ischemic heart disease (IHD), and lung cancer as well as for asthma onset in both children and adults and acute lower respiratory infections (LRI) in children [4].

In addition to epidemiological studies, a range of health impact assessment approaches have been developed for quantifying the impact of long-term PM_2.5_ exposure on mortality. In 2019, air pollution was estimated to contribute to 6.7 million deaths globally, including 4.1 million deaths attributable to ambient PM_2.5_ and 2.2 million deaths attributable to household air pollution [5, 6]. The Global Burden of Disease (GBD) 2019 study estimated that ambient particulate matter was the seventh highest risk factor globally in terms of disability adjusted life years (DALYs) and household air pollution was the tenth highest [7]. While the association between long-term exposure to PM_2.5_ and mortality has been well documented in the literature, little information is available about the impact of cycles of annual exposure increases and decreases occurring multiple times during a 20 year period, such as those due to multiple international relocations. Prior studies have used time-varying PM_2.5_ levels estimated through exposure modelling, although these studies were limited to persons that resided in one country during the study and with annual PM_2.5_ exposure levels typically below 20 µg/m^3^ [8–10]. A recent literature review of the health effects of PM_2.5_ on persons with frequent relocations identified 12 studies that reported a difference in health effects among persons who relocated and non-relocated persons [11]. These included a study that reported an increase in the hazard ratio (HR) for all-cause mortality per 10 µg/m^3^ increase in PM_2.5_ among persons who relocated within the United States, with greater effects noted among white persons (HR = 1.21; 95% CI: 1.20, 1.22) than African American persons (1.12; 1.08, 1.15) [12]. The mean change in annual ambient PM_2.5_ among movers was less than 1 µg/m^3^. A reanalysis of the US Harvard Six Cities Study reported RRs for all-cause mortality per 18.6 µg/m^3^ increment of PM_2.5_ stratified by movers (1.08; 95% CI: 0.67, 1.76) and non-movers (1.30; 1.10, 1.54), although there was limited statistical power for the findings among movers [13]. Finally, a study of congestive heart disease (CHD) deaths according to proximity to highly trafficked roads among persons in western Canada reported a lower RR of CHD death among persons who moved further away from traffic (RR = 1.14; 95% CI: 0.95, 1.37) than persons who moved closer to traffic (1.20; 1.00, 1.43) [14]. While these studies provide evidence of the impacts on mortality among persons with relocations, they do not address the impact of multiple relocations to areas with large changes in ambient PM_2.5_ concentrations.

When frequent changes of exposure are involved, the lag between the time when exposure changes and the time when the resulting health effect is evident becomes crucial. The time lag between reduced exposure and its impact on mortality (the ‘cessation lag’) has been explored by the US Environmental Protection Agency (EPA) who proposed a biphasic lag [15] in which the mortality risk evolves in two distinct phases to reach a minimum level after 20 years. This lag was informed by prior studies including a study of air pollution reductions resulting from a ban on the sale of coal in Dublin, a model developed by Leksell and Rabi of the reduction in PM_2.5_ and its impact on life expectancy based on several large cohort studies in the US and Europe, and other research on disease incidence including bronchitis, diminished lung functioning and lung cancer [16, 17]. The proposed ‘inception lag’ (i.e. the lag representing increased health risk following an increase in exposure) followed the inverse pattern of the cessation lag. Other lag functions varying in shape and duration, specifically with different non-linear slopes and timing, have been proposed for all-cause and individual cause-specific mortality [18]. They include a steep eight-year lag based on findings from the Harvard Six Cities study for PM_2.5_ exposure and all-cause mortality [19] and a 38-year triphasic lag based on smoking cessation studies and lung cancer [18, 20, 21].

The US diplomatic corps is a dynamic population with international relocations every one to six years, the timing of which depends on the diplomat’s area(s) of expertise, their US government agency and related agency staffing policies. The location of each relocation is determined by the diplomat’s agency and global staffing needs. Diplomats often have limited input into location decisions, particularly for career foreign service officers, however, preference may be given to diplomats with increasing seniority. Specific city assignments, or postings, are contingent upon medical clearance both for the employee and for their accompanying family members [22]. Time lags between exposure and exposure-related health effects are of particular concern among this population as the duration of assignment in each city is generally relatively short (often 2–3 years) and air pollution levels could vary dramatically from one assignment city to the next.

The health impacts of air pollution during international assignments for diplomats and their family members are of great concern to the US government and are one of many occupational health risks diplomats face. The US government has explored mitigation options to help reduced air pollution exposure for diplomats and their family members while working in cities with high PM_2.5_ including the filtration of air with air purifiers (APs) in offices and residences and improving home airtightness through taping or caulking windows and doorways. A study of 21 US diplomats in Kathmandu, Nepal found that the ratio of personal/ambient (P/A) PM_2.5_ was 0.32 when a moderate to high level of mitigation was already used at home. After additional high capacity APs were added in residences, the P/A ratio was halved to 0.16 [23]. There are a few other reports on the impact of using APs on indoor air quality in the US, Beijing, and Shanghai [24–26]. However, the results seem to vary among settings.

To fill the research gap on the health impact of air pollution exposures that vary over time, this study aims to develop a model to estimate the impact of frequent international relocations on mortality due to PM_2.5_ and to apply the model to US diplomats in a variety of contrasting assignment scenarios. The model explores the effects of different assumptions regarding lags between exposure and health impacts and also examines the impact of exposure mitigation measures on the mortality estimates.

## Methods

The model described in this exploratory analysis was applied to illustrative persons in three age groups (representing US diplomats and their families) in a variety of hypothetical 20-year assignment scenarios from 2000 to 2019 and was based on US mortality rates adjusted to account for time spent in locations with different levels of PM_2.5_.

### Diplomatic assignments

**Fig. 1:**
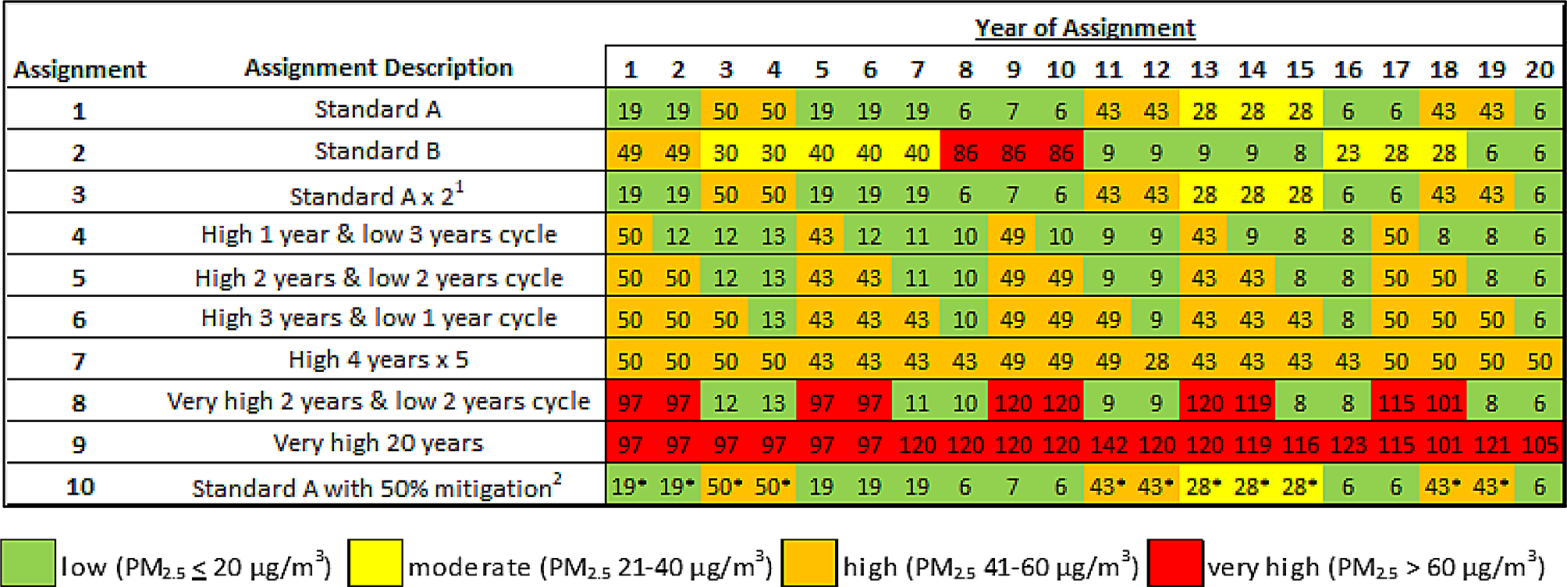
Annual mean PM_2.5_ concentration in each city for the ten assignment profiles. ^1^ The “Standard A x 2” assignment includes the Series A assignment repeated for a total duration of 40 years. ^2^ In this assignment, diplomats used air purifiers in their residences while working in cities in Africa and Asia during years indicated with an asterisk (*)

Ten hypothetical assignments were created for this analysis. The assignments and mean annual PM_2.5_ concentrations in each assignment location are shown in Fig. 1 and a list of all assignment locations is included in Supplementary Table 1. The assignments were developed to demonstrate a range of patterns of air pollution exposure and were based on typical international postings for US diplomats with occasional rotations in Washington, DC. The first two assignments, Standard A and Standard B, were based on the actual assignment histories for two former diplomats. Further variations of the Standard A assignment were used as follows: a longer assignment of 40 years from ages 25 to 64 years (Assignment 3); a cycle with one year in a highly polluted city followed by a greater number of years in a less polluted city (Assignment 4), and vice versa (Assignment 6); the same number of years of postings in highly polluted and less polluted cities (Assignments 5 and 8); highly polluted cities only (Assignment 7 and 9); and with enhanced air pollution mitigation measures including air purifiers used in personal residences and in the workplace in highly polluted cities (Assignment 10). All assignments are for 20 years, except for Assignment 3.

### Estimation of city specific PM_2.5_ exposure

All assignments were modelled for the years 2000 to 2019, except for assignment 3 (“Standard A x 2”) which covered 2000 to 2039. Each city was assigned an annual average PM_2.5_ concentration based on published estimates. In most cases, the estimated ambient PM_2.5_ concentration published in the World Health Organization’s (WHO) ambient air pollution database was used [27]. In instances where the PM_2.5_ level was not available in the WHO database for the relevant year, the annual mean PM_2.5_ for the nearest year was used. For time spent in the United States, US national and Washington, D.C. observed annual mean PM_2.5_ levels reported by the US Environmental Protection Agency were used for years 1999 to 2021 [28]. To represent previous exposure, US annual mean PM_2.5_ figures for the years 1981 to 2000 were taken from estimates by Meng et al. [29] using a chemical transport model (GEOS-Chem) with geographically weighted regression (GWR) adjustment of satellite remote sensing and ground based PM_2.5_, PM_10_ and total suspended particle (TSP) measurements, when available [29]. For years 1955 to 1980, 22.1 µg/m^3^ was used as the annual PM_2.5_ for the US as this was the estimated annual mean for 1981 reported by Meng et al. For years 2001 to 2021, US annual mean PM_2.5_ figures reported by the US Environmental Protection Agency were used in the model and for years 2022 to 2045, 8.0 µg/m^3^ was used as the annual PM_2.5_ for the US as this was the annual mean for 2021.

### Quantification of the impact on mortality

Mortality rates based on routine US statistics (see Impacted mortality rate calculations including added risk due to PM_2.5_) were used to represent those of diplomats in three age groups: (1) an older diplomat born in 1955 with assignments conducted between ages 45–64 years, (2) a young diplomat born in 1975 with assignments conducted between ages 25–44 years and (3) a child, who accompanied their diplomat parent(s) on assignments from birth to age 20 years. A standard life table method was used to estimate life expectancy and days of life lost (DLL) for each age group following each of the 10 assignments using the method described below [30]. The results were compared to those assuming the individual lived exclusively in the US over the same period.

#### GBD concentration-response functions

For each year of each assignment, we applied the GBD’s meta-regression, Bayesian, regularized, trimmed (MR-BRT) [7] concentration-response (C-R) functions to calculate the risk of PM_2.5_-related mortality in relation to the theoretical minimum-risk exposure level (TMREL) estimated by the GBD study for six outcomes: IHD, stroke, chronic obstructive pulmonary disease (COPD), LRI, type 2 diabetes mellitus, and lung cancer. The C-R functions estimate the relationship between long-term exposure to PM_2.5_ and mortality, using a non-linear exposure response model. The GBD project used data from numerous cohort studies from varied global locations with a wide variety of PM_2.5_ levels and sources.

Application: In the study, the CRFs were used to estimate the number of deaths and diseases that could be attributed to PM_2.5_ exposure globally. By combining the CRFs with global air quality data, the researchers could estimate the health burden of air pollution in different regions and countries.

In summary, the study used data-driven models to predict how air pollution impacts health worldwide, helping to understand the global burden of diseases linked to air quality.

The GBD C-R functions are based on the ambient PM_2.5_ concentration and, for IHD and stroke, vary by (5-year) age groups beginning at age 25 years. The IHD and stroke functions do not include any added risk for persons under age 25. Supplementary Fig. 1 shows the GBD C-R functions for each outcome.

#### Inception and cessation lags

As our central estimate of the lag between change in exposure and change in risk, the mortality risks calculated for each location using the GBD functions were lagged using the 20-year cessation lag published by the US Environmental Protection Agency (EPA) [31] and a 20-year inception lag assumed to be the inverse of the EPA cessation lag (Supplementary Fig. 2). The EPA 20-year cessation lag retains 70% of the risk associated with the difference in the previous year’s elevated PM_2.5_ concentration to account for short-term effects. During the next four years, the risk decreases by 12.5% per year, reaching a level of 20% risk distributed evenly over the course of the remaining 15 years with risk reduced by 1.3% per year to account for long-term effects of PM_2.5_ exposure on lung cancer mortality [21, 32]. During the first year of the (inverse) EPA inception lag, 30% of the risk is applied in the first year. During years two to five, the risk increases by 12.5% per year and during years six to twenty, the risk increases by 1.3% per year. After 20 years, the full risk is applied to the mortality rates.

To apply the lags in this analysis, we first calculated the RR of the risk in the prior location relative to the risk in the next location (i.e. the risk for the old location divided by the risk of the new location). When moving to a new location with an increase in PM_2.5_, the inception lag was applied only to the RR for the new location (location B) divided by the RR of the prior location (location A) minus 1:

$$\eqalign{& lagged\,risk,\>moved\,from\,location\,A\left( {low\,P{M_{2.5}}} \right) \cr & \,\,\,\,\,\,\,\,\,\,\,\,\,\,\,\,\,\,\,\,\,\,\,\,\,\,\,\,\,\,\,\,\,\,\,\,\,\,\,\,\,to\,B\left( {high\,P{M_{2.5}}} \right) = {{R{R_B}} \over {R{R_A}}} - 1 \cr}$$

When moving to a new location with a decrease in PM_2.5_, the cessation lag was applied to the RR for the prior location (location A) divided by the RR of the new location (location B) minus 1:

$$\eqalign{& lagged\,risk,\>moved\,from\,location\,A\left( {high\,P{M_{2.5}}} \right) \cr & \,\,\,\,\,\,\,\,\,\,\,\,\,\,\,\,\,\,\,\,\,\,\,\,\,\,\,\,\,\,\,\,\,\,\,\,\,\,\,\,\,\,\,\,\,to\,B\left( {low\,P{M_{2.5}}} \right) = {{R{R_A}} \over {R{R_B}}} - 1 \cr}$$

Risks were lagged when the ambient PM_2.5_ mean changed by *≥* 5 µg/m^3^. Supplementary Table 2 presents example calculations using an inception lag and a cessation lag. The remaining portion of the inception lag was removed from the risk calculation when a diplomat moved to a location with lower ambient PM_2.5_ while the (previous) inception lag was still evolving. Similarly, the remaining portion of the cessation lag was removed from the calculation when a diplomat moved to a location with higher ambient PM_2.5_.

#### Impacted mortality rate calculations including added risk due to PM_2.5_

US 2019 mortality rates (MRs) by age and gender were obtained for all-causes and each of the six pollution-sensitive conditions using International Classification of Diseases, 10th revision (ICD-10) codes specified in the GBD 2019 risk estimate publication [7, 33]. For each pollution sensitive condition, impacted mortality rates (iMRs) were calculated by multiplying the GBD-derived RRs by the US MRs.

The remaining, non-pollution sensitive (NPS) MR at each age was calculated by subtracting the US MR for each of the six pollution sensitive conditions from the US all-cause MR at that age. The iMRs for the six pollution sensitive conditions were added back to the NPS MR at each age to yield a new all-cause iMR.

#### Life expectancy, days of life lost and excess deaths

The probability at age ***a*** of surviving to the next year of age was calculated using the standard life table method, assuming that deaths occur at the mid-point of the year:

$$\:{survival\:probability}_{a}=\:\frac{2-{all\:cause\:iMR}_{a}}{2+{all\:cause\:iMR}_{a}}$$

Remaining life expectancy at birth, noted as *b*, was calculated by adding the life years from birth to 100 years’ age (noted as *j* in the formula) divided by the cumulative survival during year *j*:

$$\:\:{life\:expectancy}_{b}=\:\frac{\sum\:{life\:years}_{j\:}}{{cumulative\:survival}_{j}}$$

Days of life lost (DLL) were calculated by subtracting the life expectancy under each assignment from the life expectancy calculated for the same diplomat living exclusively in the US. Deaths, expressed per one million population, were calculated by multiplying the impacted all-cause mortality rate for each year of age by one million and summing the number of deaths during *j*. Deaths were calculated for male and female diplomats in each of the three age groups as well as for persons of comparable age and sex diplomats that lived exclusively in the United States. Similarly, excess deaths, expressed per million population, were calculated as the additional deaths among diplomats compared to a person of the same age and sex who lived exclusively in the United States. All key assumptions for the modelling are presented in Supplementary Table 3.

### Impact of mitigation at home and in workplace

In assignment 10, we explored the potential effects of using exposure mitigation strategies, including air purifiers and home sealing, to reduce the PM_2.5_ concentration in the homes and workplaces of diplomats working in cities in Africa and Asia. The air in most US Embassy and Consulate buildings is highly filtered and the US government provides air purifiers to US diplomats working in many cities in Africa and Asia [34]. According to the results from our previous personal monitoring study among US diplomats in Kathmandu, Nepal, the ratio of mean *personal* PM_2.5_ exposure to mean *ambient* PM_2.5_ was reduced by 50% (from 0.32 to 0.16) following the addition of enhanced mitigation in diplomats’ residences [23]. These findings were applied to the “Standard A with mitigation assignment” by using 50% of the city-year specific PM_2.5_ exposure level during international assignments in Africa and Asia.

### Sensitivity analysis

We conducted eight sensitivity analyses to examine the sensitivity of the model results to key assumptions/parameters using the Standard A assignment. Variation 1 used US mortality statistics from the top 5% of US counties according to total household income [35]) to reflect the fact that the baseline mortality for diplomats is likely to be lower than the US average population. To test the sensitivity of the model to the chosen lag structures, Variations 2 to 6 used alternate lag structures applied to the GBD C-R functions: Variation 2 used a steep eight-year lagapplied to COPD and LRI only (no change in other four causes); Variation 3 used a longer 38-year triphasic lag for lung cancer only (no change in other five causes); Variation 4 applied only the cessation lag (i.e. no inception lag, assuming the full effect from the first year in a new location with higher PM_2.5_ level); Variation 5 applied only the inception lag (i.e. no cessation lag, assuming the increased mortality risk associated with time spent in a location with high PM_2.5_ was discontinued upon leaving that location); and Variation 6 applied no lagged effects. Supplementary Fig. 2 includes plots of the lags used in variations 2 (short lag applied to COPD and LRI) and 3 (long lag applied to lung center). To assess the effects of uncertainty in the C-R functions, Variations 7 and 8 used the upper and lower limits of the 95% confidence intervals around the GBD functions.

## Results

**Fig. 2:**
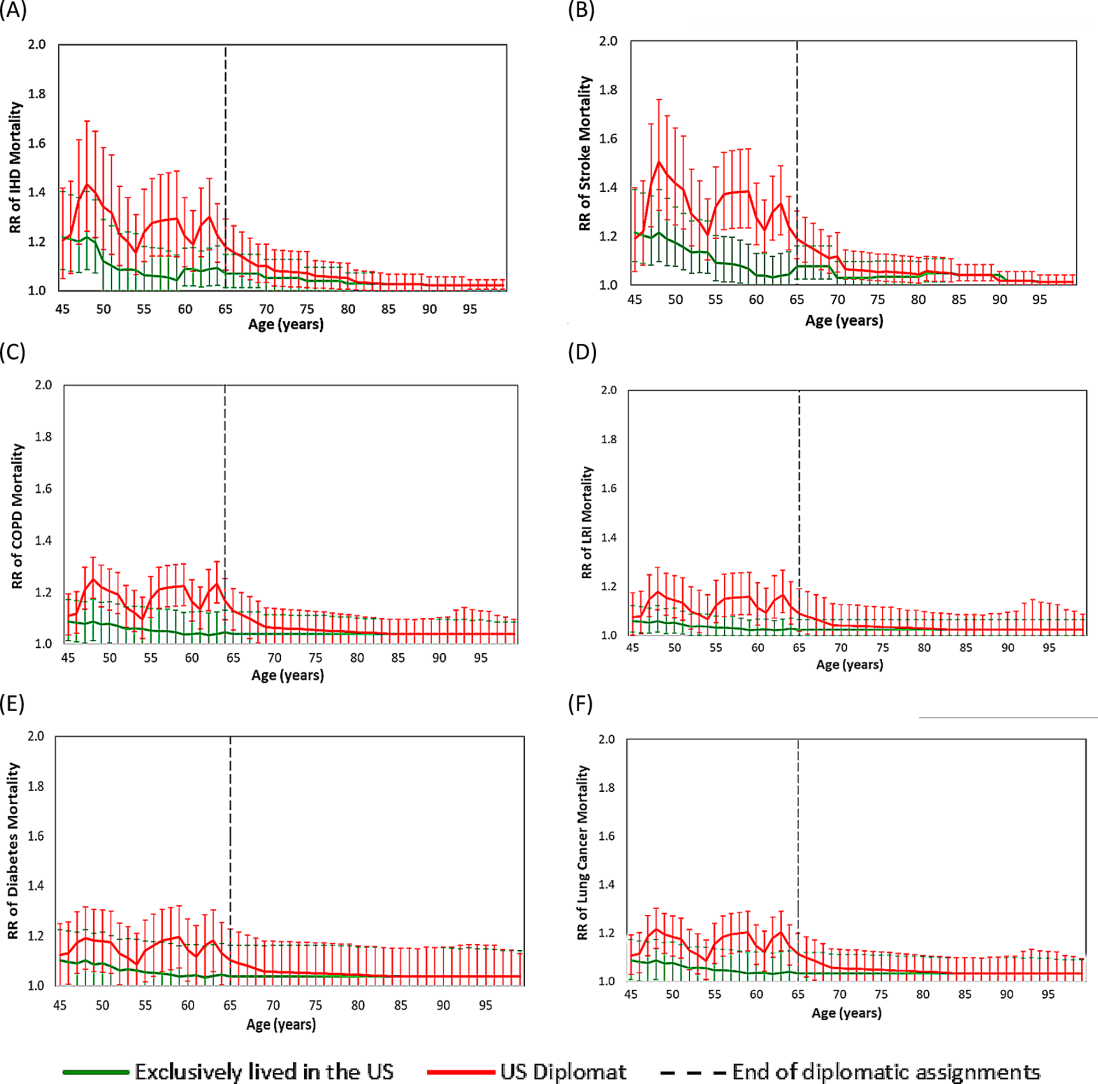
Increase in relative risk of mortality for six pollution-sensitive conditions for an older diplomat in the Standard A assignment in comparison to an America living exclusively in the US during their lifetime. **A** IHD. **B** Stroke. **C** COPD. **D** LRI. **E** Type 2 diabetes mellitus. **F** Lung cancer.

The cause-specific lagged increases in the relative risks (RR) of mortality due to stroke, IHD, COPD, lung cancer, LRI, and type 2 diabetes for an older diplomat who began the Standard A assignment at age 45 years are included in Fig. 2. There were no lags applied prior to the beginning of the diplomatic assignment and an inception lag was applied during years 1–4, 11–14, and 18–19 of the Standard A assignment when the diplomat lived in a city with ambient annual PM_2.5_ that was at least 5 µg/m^3^ greater than the prior city location and a cessation lag was applied during years 5–10, 15–17, and 20 when the diplomat moved to a city with ambient annual PM_2.5_ at least 5 µg/m^3^ less than the prior city’s PM_2.5_ annual mean. A cessation lag was also applied at the conclusion of the diplomatic assignment from age 65–84 years.

After the older diplomat began the Standard A assignment at age 45, among the six pollution-sensitive conditions, the greatest difference between RRs for the diplomat compared to the RR for the person who lived exclusively in the US was observed for stroke during the fifteenth year of the assignment, during the third and final year working in Bangkok, Thailand with an RR of 1.39 (95% CI: 1.23, 1.57) and mean annual PM_2.5_ of 27 µg/m^3^. During the same year, the RR of stroke for a person living in the US was 1.07 (1.0, 1.17) and mean annual PM_2.5_ of 9 µg/m^3^. During the course of the 20-year Standard A assignment, the RRs of mortality for each of the six pollution sensitive conditions have three distinct peaks, each occurring at the conclusion of a series of years with consecutively increasing ambient PM_2.5_ levels. The pollution sensitive condition with the second greatest annual difference in the RR of mortality during the Standard A assignment for a diplomat compared to a person living in the US was for IHD, with an RR of 1.29 (95% CI: 1.14, 1.49), during the third and final year working in Bangkok when the mean annual PM_2.5_ was 27 µg/m^3^ (Fig. 2A). During the same year, the RR of IHD for a person living in the US was 1.04 (95% CI: 1.00, 1.15) and mean annual PM_2.5_ of 9 µg/m^3^, and the confidence intervals (CIs) for the diplomat and the person of comparable age living in the US overlap. The RRs of mortality for IHD and stroke are elevated before the older diplomat began the Standard A assignment at age 45 and these RRs are reflective of the GBD C-R functions based on US PM_2.5_ levels. Supplementary Fig. 3 shows the pollution sensitive condition iMRs for an older diplomat in the Standard A assignment.

**Fig. 3:**
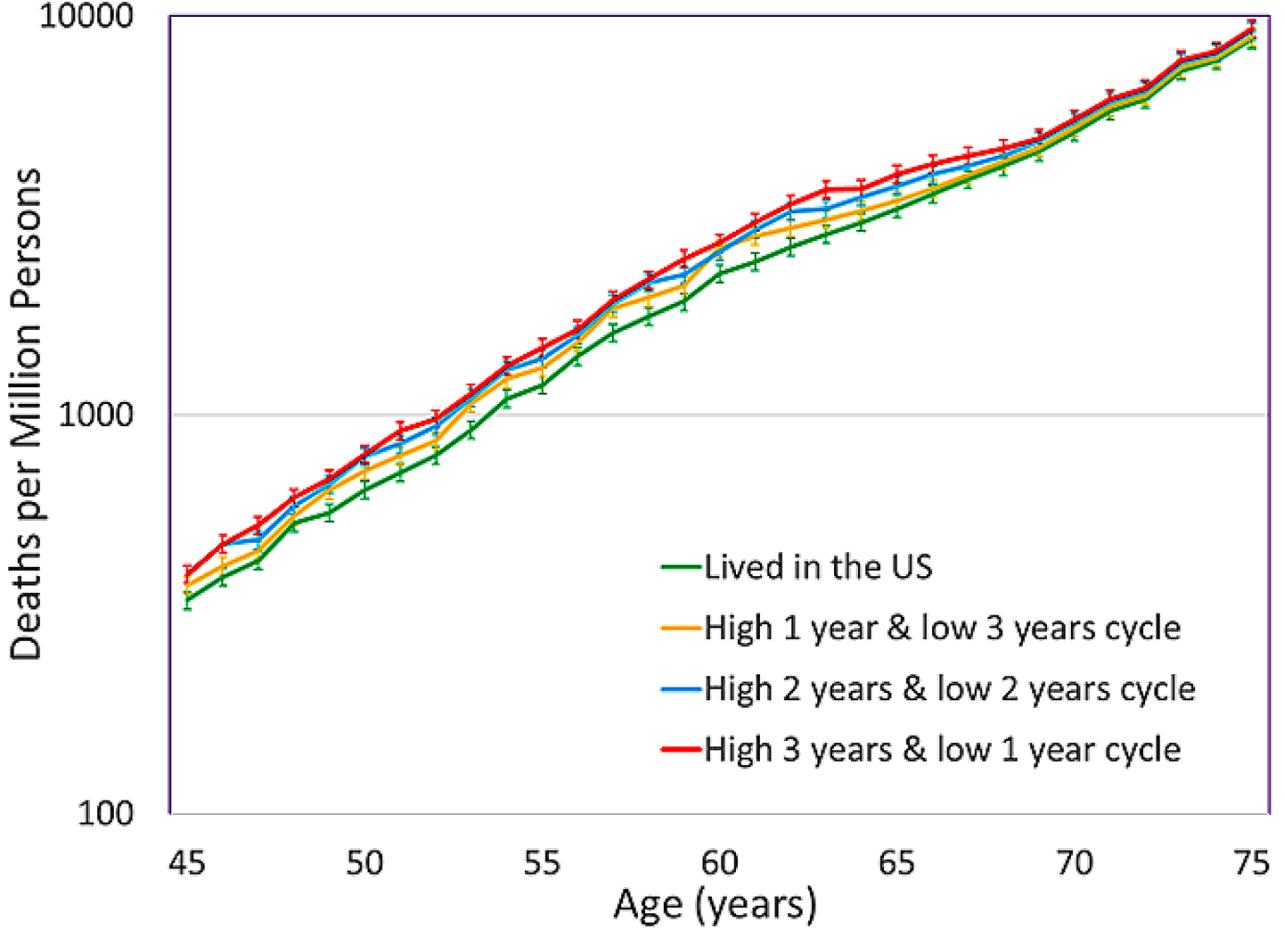
Age-specific deaths per million for female older diplomats in three assignments

Figure 3 shows the deaths per one million persons due to all-causes, that incorporates the individual pollution sensitive iMRs for female older diplomats in various assignments. The assignment “High 3 years & low 1 year cycle” has the highest impacted mortality rate each year compared to the other assignments for most of the 20-year assignment period, from ages 45–64, although the CIs for the three assignments overlap in 19 of the 20 years of diplomatic postings. The CIs for the “High 3 years & low 1 year cycle” and the “High 2 years & low 2 years cycle” assignments do not overlap with the CIs for the US iMRs. However, the CIs for the assignment with the least number of years in a location with high PM_2.5_, “High 1 year & low 3 years cycle”, overlap with the CIs for the US iMRs during 19 of the 20 years of the diplomatic assignments. Beginning at age 65 after the completion of the assignments, the iMRs for the three assignments gradually decline to the US MR.

During the Standard A and Standard B assignments, older diplomats had a greater number of excess deaths due to international assignments than young diplomats and children accompanying a parent on diplomatic assignments (Table 1). During the Standard A assignment, older diplomats (12,246.8 deaths per one million males, 6,640.7 deaths per one million females) had 8 times as many excess deaths as young diplomats (1,527.9 deaths per one million males, 708.6 deaths per one million females) and more than a 600-fold increase in excess deaths compared to a child accompanying their parent(s) on a diplomatic assignment (20.1 deaths per one million males, 12.1 deaths per one million females). Among the assignments examined, older diplomats living in one city with very high PM_2.5_ for 20 years had the highest number of excess deaths due to international assignments (49,140.4 deaths per one million males, 28,758.3 deaths per one million females), followed by living in a series of high PM_2.5_ cities in the assignment “High 4 years x 5” (29,658.2 deaths per one million males, 16,517.3 deaths per one million females). Among older diplomats in assignments with one, two or three of every four years living in locations with high PM_2.5_ (PM_2.5_ annual mean between 41 and 60 µg/m^3^), the highest number of excess deaths were found during assignments with the highest number of years in cities with high PM_2.5_ locations, including the assignments “High 3 years & low 1 year cycle” (22,120.1 deaths per one million males, 12,329.7 deaths per one million females), “High 2 years & low 2 years cycle” (15,496.9 deaths per one million males, 8,555.1 deaths per one million females) and “High 1 year & 3 low years cycle” (10,009.2 deaths per one million males, 5,409.8 deaths per one million females).

**Table 1:** Number of excess deaths^1^ per one million persons for each diplomatic assignment^2^

		Older diplomat		Young diplomat		Child	
Assignment	Assignment description^3^	Excess deaths, male	Excess deaths, female	Excess deaths, male	Excess deaths, female	Excess deaths, male	Excess deaths, female
1	Standard A	12,246.8	6,640.7	1,527.9	708.6	20.1	12.1
2	Standard B	10,568.4	5,623.3	1,341.8	562.7	43.7	27.9
3	Standard A x 2	n/a	n/a	14,370.6	7,896.7	n/a	n/a
4	High 1 year & low 3 years cycle	10,009.2	5,409.8	1,171.2	536.3	40.7	26.7
5	High 2 years & low 2 years cycle	15,496.9	8,555.1	2,050.4	975.8	60.3	37.9
6	High 3 years & low 1 year cycle	22,120.1	12,329.7	3,007.8	1,466.6	75.7	44.4
7	High 4 years x 5	29,658.2	16,517.3	7,695.6	5,211.2	86.8	53.1
8	Very high 2 years & low 2 years cycle	24,988.7	14,514.8	3,304.8	1,678.9	108.5	70.7
9	Very high 20 years	49,140.4	28,758.3	6,332.1	3,321.5	163.5	103.8
10	Standard A with mitigation^4^	4,119.6	2,117.3	406.0	148.8	-1.8	-0.9

^1^The number of excess deaths per year *i* is calculated by subtracting the number of deaths during yar i per 1 million persons of the same age and sex living exclusively in the US from the number of deaths during ear i per 1 million diplomats completing a diplomatic assignment. The number of excess deaths listed on this table is the sum of excess deaths for each year from birth to age 100

^2^See Supplemental Table 1 for a list of cities and number of years in each city during the 10 assignments and see Fig. 1 for the annual mean PM_2.5_ levels used in the calculations

^3^Ambient PM_2.5_ level descriptions include low (PM_2.5_<20 µg/m^3^), moderate (PM_2.5_ 21–40 µg/m^3^), high (PM_2.5_ 41–60 µg/m^3^) and very high

(PM_2.5_>61 µg/m^3^)

^4^ In this assignment, diplomats used air purifiers in their residences while working in cities in Africa and Asia

**Fig. 4:**
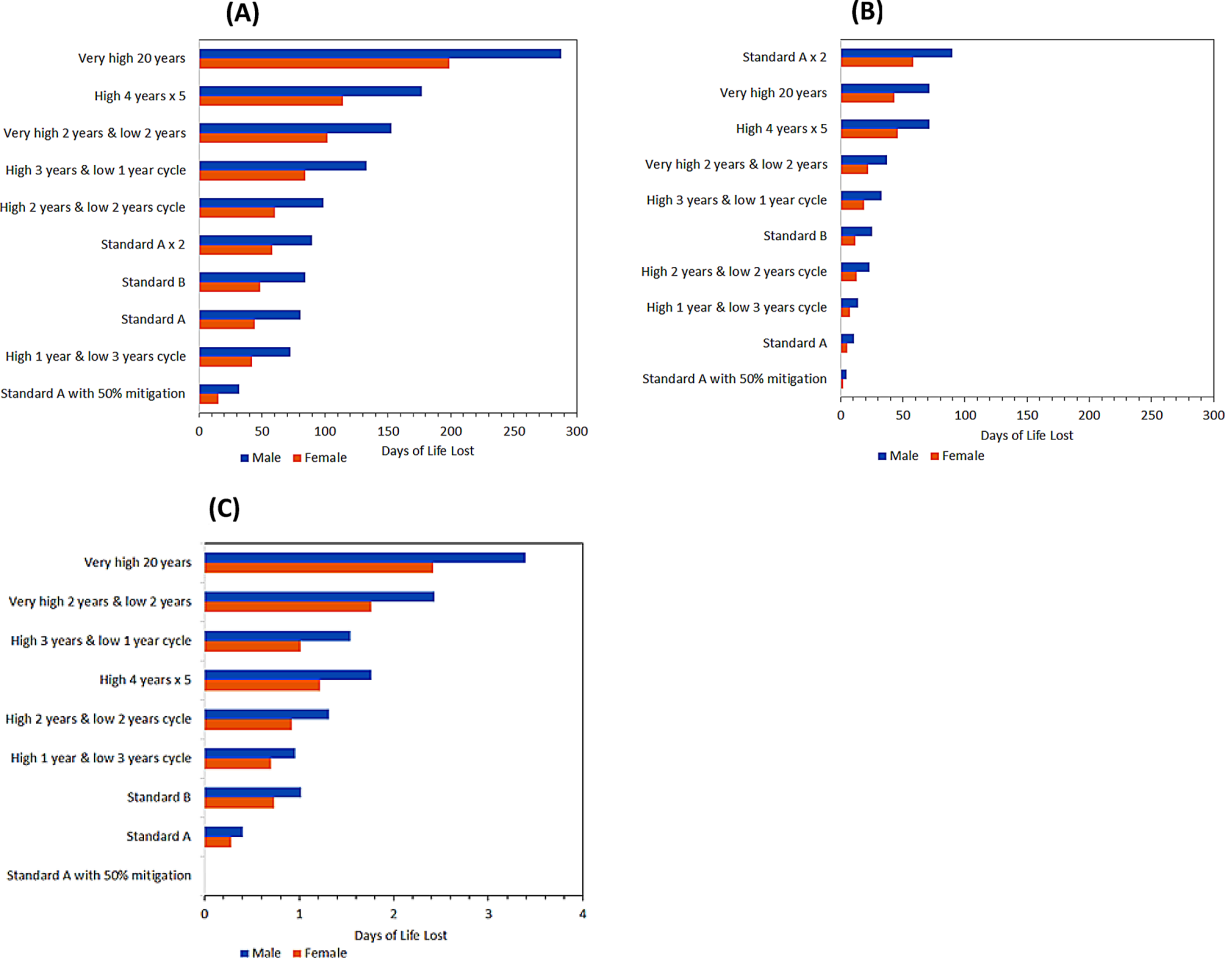
Days of life lost associated with assignments for diplomats in three age groups. **A** Older diplomat. **B** Young diploma©. **C** Child

Older diplomats had greater DLL than young diplomats in the Standard A assignment (80.4 DLL vs. 10.4 DLL) and in the Standard B assignment (84.1 DLL vs. 25.0 DLL) (Fig. 4). Similar trends were found for females, although the magnitude of impact was smaller than that of males. Among older male diplomats, the greatest DLL were observed during the assignment in one city with very high PM_2.5_ for 20 years, “Very high 20 years” (287.8 DLL), followed by serving in four cities with high PM_2.5_ for a total of 20 years, “High 4 years x 5” (176.9 DLL), and an equal number of years in cities with very high PM_2.5_ and low PM_2.5_, “Very high 2 years & low 2 years” (152.8 DLL). Among older male diplomats completing assignments with one, two or three of every four years in a city with high PM_2.5_, the “High 3 years & 1 low year cycle” (132.9 DLL) assignment had the highest number of DLL among older male diplomats. The magnitude of DLL decreased as the number of years in cities with high PM_2.5_ per four year cycle decreased to a low of 72.4 DLL while serving in the assignment with one year in a city with high PM_2.5_,“High 1 year & low 3 years cycle”. A male completing a 40-year assignment, “Standard A x 2” from ages 25–64 years (89.7 DLL) had only slightly higher DLL than a male completing the Standard A assignment for 20 years from ages 45–64 (80.4 DLL).

**Fig. 5:**
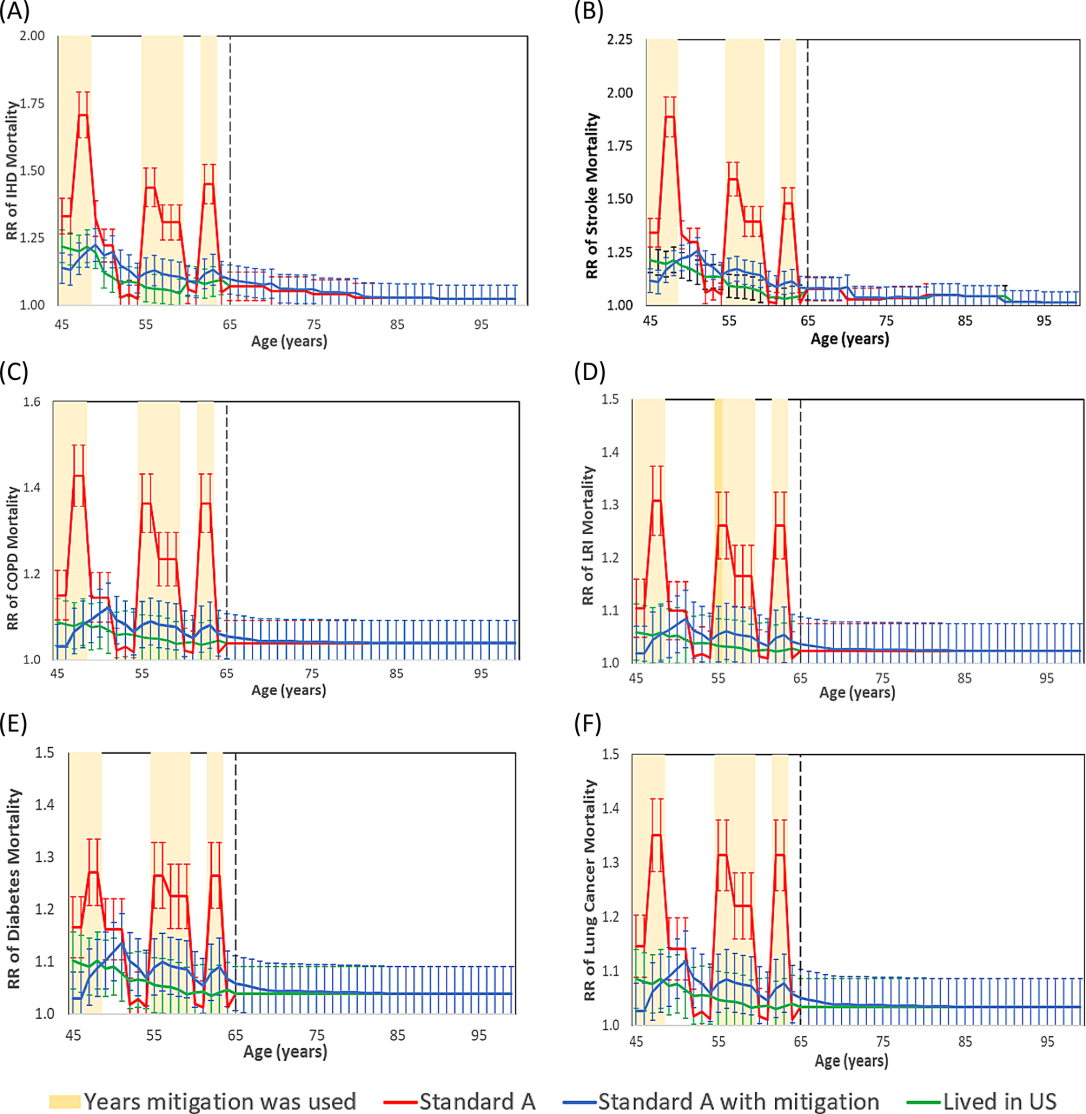
RR of mortality due to PM_2.5_ for the Standard A assignment with and without mitigation. (**A**) IHD, (**B**) Stroke, (**C**) COPD, (**D**) LRI, (**E**) Type 2 Diabetes and (**F**) Lung Cancer.

**Fig. 6:**
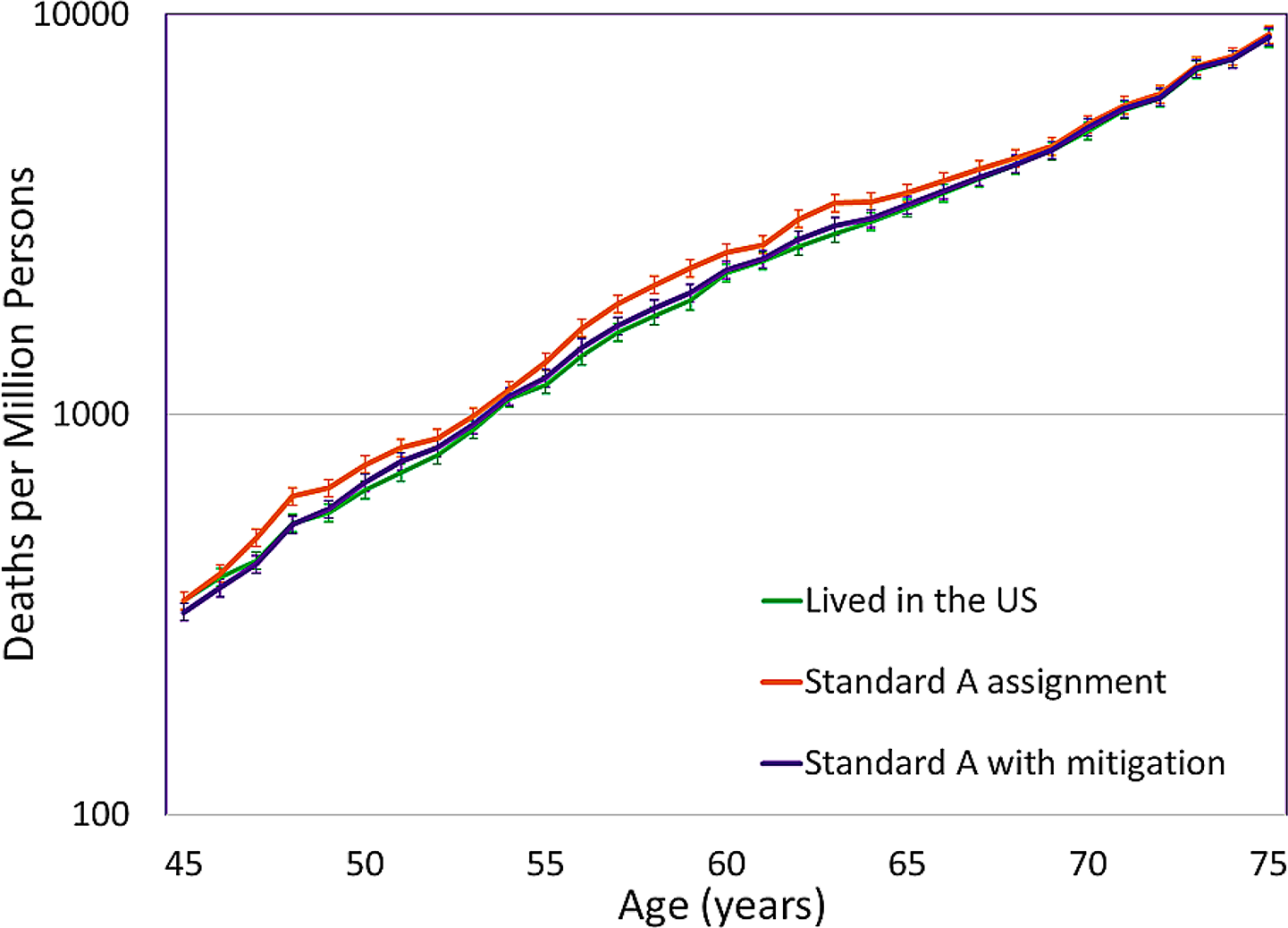
Deaths per million female older diplomats in the Standard A assignment without mitigation

The RRs of cause-specific mortality for an older diplomat in the Standard A assignment with mitigation (Fig. 5) were much closer to that of a person of the same age and sex living in the US than in the Standard A assignment without mitigation. Similarly, the number of deaths per million older female diplomats in the Standard A assignment with mitigation (Fig. 6) were much closer to that of a person of the same age and sex living in the US than in the Standard A assignment without mitigation. Examining the number of excess deaths among male diplomats for the Standard A assignment with mitigation compared to the Standard A assignment without mitigation, excess deaths decreased by 66% for older diplomats (from 12,246.8 to 4,119.7 deaths per million), 73% for young diplomats (from 1,527.9 to 406.0 deaths per million) and 49% for children of diplomats (from 3.5 to 1.8 deaths per million) (Table 1). Among males the DLL decreased by 60% for older diplomats (from 80.4 to 32.0 DLL), 55% for young diplomats (from 10.4 to 4.7 DLL) and results were similar in both scenarios for children (0.4 and − 0.1 DLL).

**Fig. 7:**
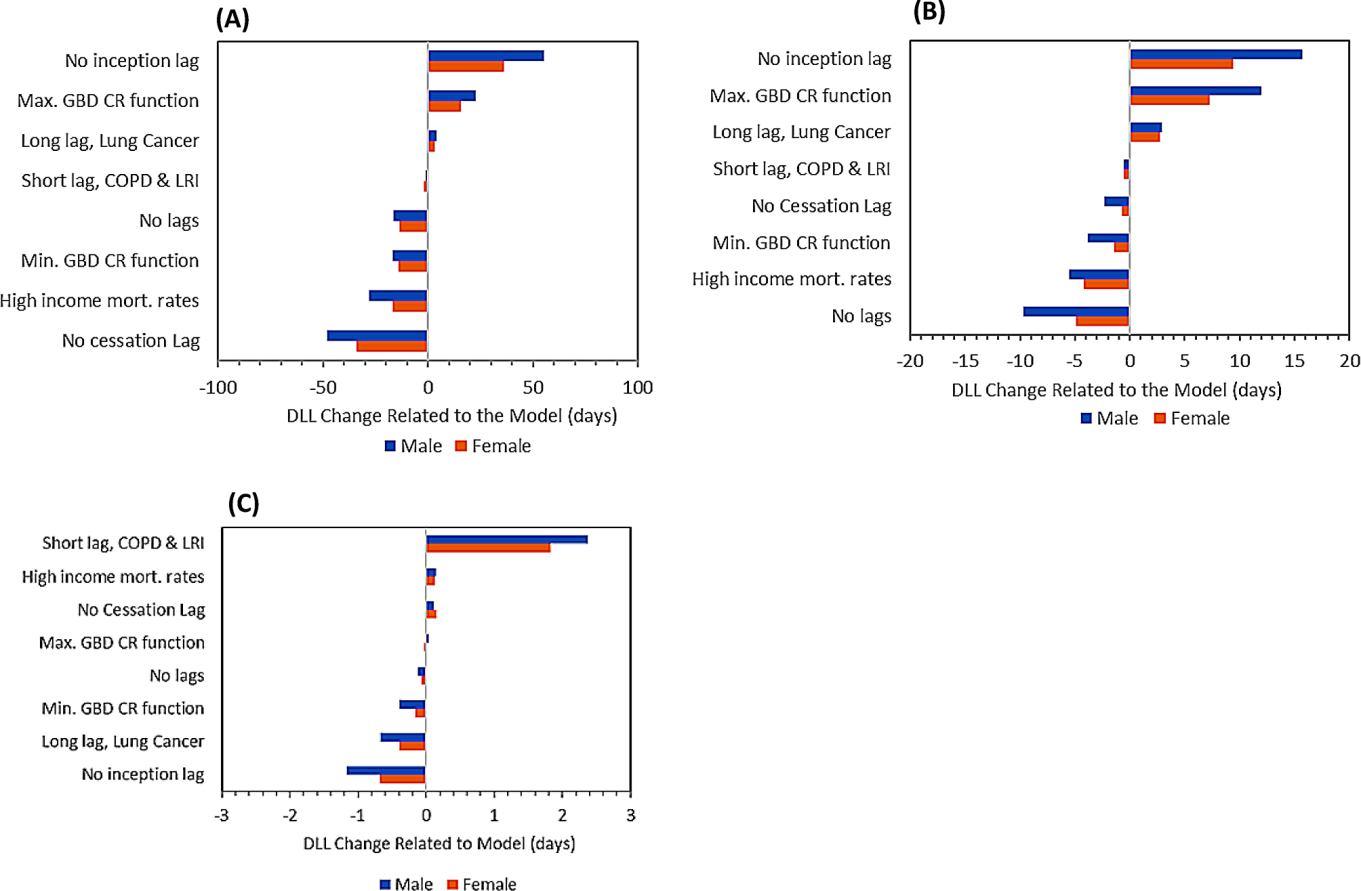
Change in the DLL in sensitivity analysis for diplomats in three age groups. (**A**) Older diplomat, (**B**) Young diplomat and (**C**) Child.

The differences in DLL for the specified sensitivity analysis options were quantified by subtracting the DLL from the main model reported above: the differences in DLL ranged from − 48.0 to + 55.1 for older diplomats, − 9.7 to + 15.7 for young diplomats and − 1.2 to + 2.4 for children (Fig. 7). Removing the inception lag had the largest impact on the DLL among the investigated model variations for older diplomats (+ 55.2 (+ 68%) DLL male, + 36.4 (+ 76%) DLL female) and younger diplomats (+ 15.7 (+ 151%) DLL male, + 9.4 (+ 90%) DLL female). Using the upper CI of the C-R function also resulted in an increase in DLL both among older diplomats (+ 22.5 (+ 28%) DLL male, + 15.6 (+ 33%) DLL female) and young diplomats (+ 12.0 (+ 115%) DLL male, + 7.3 (+ 70%) DLL female). Using the short lag on COPD and LRI and, separately, the long lag on lung cancer, both had small impacts on the DLL for older and young diplomats. Model variations applied to older diplomats resulted in a decrease in DLL: removing lags from the model (-16.3 (-20%) DLL male, -13.9 (-29%) DLL female), using the minimum C-R function (-17.1 (-21%) DLL male, -14.2 (-30%) DLL female), using high income mortality rates (-28.2 (-35%) DLL male and female) and removing the cessation lag (-48.0 (-59%) DLL male, -34.0 (-71%) DLL female). Application of the short lag to COPD and LRI suggested the largest possible impacts on DLL for children (+ 2.9 (+ 431%) DLL male, female + 1.8 (+ 465%) DLL female). All other model variations resulted in less than 1 DLL gained or lost. Use of high income US baseline mortality statistics, instead of mortality statistics for the entire US population, resulted in a slight gain in DLL (+ 0.7 (+ 103%) DLL male, (+ 0.4 (+ 95%) DLL female) as did removal of the cessation lag for all six pollution sensitive conditions (+ 0.7 (+ 100%) DLL male, + 0.5 (+ 100%) DLL female). Use of the 20-year inception and cessation lags yielded results that were roughly midway between the results when other elements of the model were changed.

## Discussion

We developed a novel approach to estimate the impact on mortality due to local PM_2.5_ concentrations given assignments with frequent relocations to areas of varying PM_2.5_. A set of systematically designed 20-year diplomatic assignments were used to demonstrate the impacts of the assignment patterns of city-specific annual mean PM_2.5_ on the health of US diplomats. The key findings include that age at the time of relocation is critically important in determining the impact of air pollution on mortality, with greater impacts found for relocations amongst older diplomats than for young diplomats or the child of a diplomat (due to greater underlying mortality rates in older age groups). The mortality impacts decreased when assignments in high PM_2.5_ cities were followed by assignments in low PM_2.5_ cities and using room APs and improving home airtightness in polluted cities may have the potential to reduce the impacts considerably. According to the sensitivity analysis, the estimated health impacts were more sensitive to the choice of inception and cessation lags than to the PM_2.5_ exposure level, C-R functions, or baseline mortality. Removal of the inception lag increased the DLL by 68% and 76% for male and female older diplomats compared to the original model including both lags, while removal of the cessation lag reduced the DLL by 59% and 71%.

This study demonstrated a new indirect method using WHO air quality data and GBD C-R functions to quantify the lifetime health impacts of air pollution exposure that frequently changes for a highly mobile population with relocations between global cities. There are several notable features in the model. First, comparison with baseline mortality using the life table analysis enables calculation of DLL considering the time when death occurs during the life course. This adds value to estimates based on the number of deaths which are commonly reported by previous quantitative risk assessments such as the GBD study [7]. Second, the model enables incorporation of various time lag functions in terms of inception and cessation, depending on the target health outcome. The dramatic changes in PM_2.5_ exposure among the US diplomatic population highlight a paucity of evidence on the cause-specific delayed effects of changes in PM_2.5_ exposure.

Our exploratory study also has several limitations and methodological challenges. First, there was limited availability of annual mean PM_2.5_ data for cities used in the assignment scenarios, particularly for years prior to 2010. The use of PM_2.5_ annual mean data from several years before or after the year of interest may result in misclassification of exposure. This could underestimate the exposure in many cities because PM_2.5_ was generally higher in earlier years when it was not commonly measured. Future development of the method would therefore benefit from consistent, routine ambient PM_2.5_ monitoring across global cities, particularly in low and middle income countries. However, the accuracy of the PM_2.5_ data is a lesser concern as the purpose of this study was to demonstrate changes in mortality associated with illustrative variations in PM_2.5_ levels and not to directly attribute it to time spent in any particular city. Second, the model did not take into account the different, but highly uncertain, nature of PM_2.5_ sources in each global location and the impact that PM_2.5_ from different sources may have on mortality [36]. Indeed, our study included cities where the majority of PM_2.5_ can be attributed to emissions from soils, plants, and dust (Riyadh and Dakar) and cities with high levels of PM_2.5_ due to burning biofuels for domestic energy use (Bangkok and Kathmandu). Third, using estimates of city-wide PM_2.5_ exposure may not provide an accurate representation of exposure for diplomats as they may spend more time in less polluted areas in the city as well as using Aps in their workplace and homes. Fourth, our method likely underestimates impacts in children because the GBD C-R functions were not designed to be used to estimate children’s health risks and, in particular, the GBD 2019 study did not include C-R functions for IHD or stroke occurring in persons under 25 years age because the incidence of IHD and stroke in persons under 25 years age is very low and unlikely to be impact by PM_2.5_. It should also be noted that our model included only mortality and the effects of air pollution on children may be more pronounced in terms of morbidity, such as exacerbation of asthma. Fifth, we used baseline mortality risks for the US general population to represent the baseline mortality risks for US diplomats which may not be entirely representative for diplomats’ health conditions, as diplomats are likely to be healthier than the general population due to better nutrition and increased access to medical care. In order to address this issue, we explored the effects of limiting the baseline mortality rates to those with higher socioeconomic status (SES) in the sensitivity analysis. However, C-R functions may also be different for the higher SES population as modification of PM_2.5_ effects on mortality by SES is very often observed in cohort studies [37–39]. Sixth, the set of 10 illustrative assignments used in this study does not cover the wide range of realistic assignment patterns for US diplomats. Application of this model to a greater number of assignments using actual human resources data for US diplomats would provide realistic total mortality burdens among the US diplomatic population. Seventh, the use of mitigation activities including APs at home and improved home tightness in cities with high PM_2.5_ assumed a 50% reduction in PM_2.5_ exposure in those cities, based on results of our previous Kathmandu study [23]. As the reduction of the outdoor PM_2.5_ level depends on the housing structure in each local setting in a different city, this is a very rough estimate and a universal assumption applied to a range of cities. While the results associated with mitigation suggested beneficial effects for all age groups, the reported DLLs should be interpreted with caution. Nonetheless, this example illustrates the possible health benefits, in terms of the reduction of DLLs, through the use of mitigation at home and in the workplace in highly polluted cities. An additional limitation of the analysis is the exclusive reliance on one set of exposure functions, notably the GBD’s C-R functions for PM_2.5_. While the sensitivity analysis examined the impact on mortality based on the upper and lower confidence intervals of the C-R functions, a tenth sensitivity analysis could have included the use of an alternative exposure function, such as 1.21 hazard ratio for all-cause mortality per 10 µg/m^3^ increase in PM_2.5_, identified by Awad et al. among white Americans who relocated within the US [12]. Finally, this research did not examine the impact on mortality of other factors that change when diplomats relocate including changes in their diet, access to clean potable water and food sources, nor changes in mortality due to motor vehicle accidents, violent crimes, or natural disasters [40–42]. Future research could benefit from including the impact of these exposures by location on diplomats’ mortality estimations, in addition to examining the PM_2.5_-related impacts on mortality.

The modelling strategy proposed here is unique and very few prior studies are directly comparable to the modeling strategy proposed here. A study conducted among US military families who experienced frequent relocations within the US reported that respiratory hospitalizations among children aged 2–5 years increased among those living in cities with high ozone (O_3_) levels, although the study suffered from limited power when the analysis was confined to movers [43]. The study focused on US domestic relocations and did not involve dramatic changes in air pollution exposure. Several studies have quantified the health impacts of mitigation measures to reduce air pollution exposure. A UK modeling study found that lifetime use of Aps at home may increase life expectancy by 138 and 120 days or more for males and females, respectively [44]. Like ours, the study used life table methods and GBD C-R functions for IHD, stroke, COPD, LRI and lung cancer, although neither inception nor cessation lags were applied in the model, which is less crucial for minimal changes in exposure. A US modeling study evaluated the mortality-related benefits and costs of improvements in particle filtration in US homes and commercial buildings accounting for time spent in various environments as well as activity levels and associated breathing rates. The results indicated that the use of portable Aps in homes in the US could be a cost-effective strategy to reduce particle-related mortality [45]. While our results with mitigation measures were encouraging, the model did not address any detrimental effects of improved home airtightness, including possibly increased indoor PM_2.5_, carbon dioxide and other indoor pollutants [46, 47].

The findings from this study provide useful information to support decision making to reduce health risks for people with frequent international relocations including the diplomatic corps and other professional groups whose PM_2.5_-related mortality risk and life expectancy may be affected by overseas assignments. Our results suggest that, where possible, hiring agencies and employees may want to consider scheduling work in cities with high PM_2.5_ at the beginning of the career rather than at older ages, when baseline mortality rates increase steeply with age. After completion of a posting in a city with high PM_2.5_, hiring agencies and employees may also want to request that their next posting be in a city with low PM_2.5_, if possible. It should be noted that there are many important factors in determining staffing assignments including the suitability for available positions, amount of training and expertise needed in various positions, as well as other personal and family health and safety considerations. Although ambient PM_2.5_ could impact diplomats’ health, it is only one of many important factors that employers may want to consider when determining global staffing assignments and the duration of assignments. Our previous study in Kathmandu suggested that the use of high capacity air purifiers and improvement of building airtightness greatly reduced PM_2.5_ personal exposure [23]. Additional research is needed to better understand the association between personal and ambient PM_2.5_ in different global settings to estimate the impact of mitigation more precisely on PM_2.5_-related mortality. The application in this model of an indicative reduction rate of PM_2.5_ exposure from the Kathmandu study suggested that potential health benefits could be achieved from these mitigation measures. In reality, there are inequalities in access to such expensive mitigation measures across the globe, especially in LMICs. In addition, the use of Aps may not be an ideal solution given concerns about climate change and planetary health.

## Conclusions

We developed a novel health impact model to estimate the effect of lifetime exposure to changing PM_2.5_ levels on mortality for individuals with regular international relocations, by applying published C-R functions with inception and cessation lags. The application of the model to US diplomats in various assignments suggested that an increased number of years living in high PM_2.5_ cities resulted in an elevated mortality risk, with greater health impacts in older diplomats than young diplomats or their children because of their greater underlying mortality rates for conditions sensitive to air pollution. Alternating assignments, when possible spending a few years in a high PM_2.5_ city followed by a year or more in a city with a lower PM_2.5_ concentration may help to reduce the additional risk of mortality due to PM_2.5_. Our results also suggest that the use of air purifiers and improved home airtightness may help mitigate health burdens due to exposure to ambient PM_2.5_. The choice of inception and cessation lags is critical for the magnitude of the estimated mortality burdens and, as such, further research on the delayed effects of PM_2.5_ exposure on cause-specific mortality is required to improve model estimates.

## Electronic supplementary material

Below is the link to the electronic supplementary material.

Supplementary Material 1

## Data Availability

No datasets were generated or analysed during the current study.

## Abbreviations

AP: Air purifier
CHD: Congestive heart disease
COPD: Chronic obstructive pulmonary disease
C-R: Concentration response function
DALY: Disability adjusted life year
DLL: Days of life lost
EPA: US Environmental Protection Agency
GBD: Global Burden of Disease
HR: Hazard ratio
IHD: Ischemic heart disease
I/O: Ratio of indoor PM_2.5_ to outdoor PM_2.5_
LRI: Lower respiratory infection
MR-BRT: Meta-regression, Bayesian, regularized, trimmed
P/A: Ratio of personal PM_2.5_ to ambient PM_2.5_
PM_2.5_: Particulate matter smaller than 2.5 μm
RR: Relative risk
TRAP: Traffic-related air pollution
TMREL: Theoretical minimum-risk exposure level
YLL: Years of life lost
