# Supplementary material for: The impact of changing exposure to PM2.5 on mortality for US diplomats with multiple international relocations: a modelling study
Source: Environ Health. 2024 Oct 22;23:89. doi: 10.1186/s12940-024-01127-6 (PMC11495087; doi:10.1186/s12940-024-01127-6)
Supplement: Supplementary file 1 — Supplementary Material 1 [file 12940_2024_1127_MOESM1_ESM.docx]

**Manuscript Title: The impact of changing exposure to PM_2.5_ on mortality for US diplomats with multiple international relocations: A modelling study**

Leslie Edwards, James Milner, Paul Wilkinson, Ai Milojevic

London School of Hygiene and Tropical Medicine, London, UK

Supplementary Material

**Supplementary Table 1**: Ten diplomatic assignments with the city and number of years in each city indicated

**Supplementary Table 2:** Example calculations of the impacted MR for COPD using inception and cessation lags

**Supplementary Table 3**: Assumptions of the model and the sensitivity analysis

**Supplementary Figure 1**: Global Burden of Disease 2019 concentration-response (C-R) functions for 6 pollution-sensitive conditions

**Supplementary Figure 2**: Inception (A) and cessation (B) lags used in the model and sensitivity analysis

**Supplementary Figure 3**: Mortality rates for six pollution-sensitive conditions, older diplomat, Standard A assignment

**Supplementary Table 1: Ten diplomatic assignments with the city and number of years in each city indicated^1^.**

| **Assignment** | **Assignment Description** | **Assignment locations (number of years in each location)** |
| --- | --- | --- |
| 1 | **Standard A** | Manila (2 years), Riyadh (2), Munich (2), San Francisco (3), Kathmandu (2), Bangkok (3), Washington, DC (2), Dakar (2), Washington, DC (1) |
| 2 | **Standard B** | Yaoundé (2), Tirana (2), Amman (3), Dhaka (3), Washington, DC (5), Hanoi (3), Stockholm (2) |
| 3 | **Standard A x 2^1^** | Manila (2 years), Riyadh (2), Munich (2), San Francisco (3), Kathmandu (2), Bangkok (3), Washington, DC (2), Dakar (2), Washington, DC (1), Manila (2), Riyadh (2), Munich (2), San Francisco (3), Kathmandu (2), Bangkok (3), Washington, DC (2), Dakar (2), Washington, DC (1) |
| 4 | **High 1 year & low 3 years cycle** | Riyadh (1), Washington, DC (3), Kathmandu (1), Washington, DC (3), Yaoundé (1), Washington, DC (3), Dakar (1), Washington, DC (3), Riyadh (1), Washington, DC (3) |
| 5 | **High 2 years & low 2 years cycle** | Riyadh (2), Washington, DC (2), Kathmandu (2), Washington, DC (2), Yaoundé (2), Washington, DC (2), Dakar (2), Washington, DC (2), Riyadh (2), Washington, DC (2) |
| 6 | **High 3 years & low 1 year cycle** | Riyadh (3), Washington, DC (1), Kathmandu (3), Washington, DC (1), Yaoundé (3), Washington, DC (1), Dakar (3), Washington, DC (1), Riyadh (3), Washington, DC (1) |
| 7 | **High 4 years x 5** | Riyadh (4), Kathmandu (4), Yaoundé (4), Dakar (4), Riyadh (4) |
| 8 | **Very high 2 years & low 2 years cycle** | New Delhi (2), Washington, DC (2), New Delhi (2), Washington, DC (2), New Delhi (2), Washington, DC (2), New Delhi (2), Washington, DC (2), New Delhi (2), Washington, DC (2) |
| 9 | **Very high 20 years** | New Delhi (20) |
| 10 | **Standard A with mitigation^2^** | Manila* (2 years), Riyadh* (2), Munich (2), San Francisco (3), Kathmandu* (2), Bangkok* (3), Washington, DC (2), Dakar* (2), Washington, DC (1) |

^1^ Each of the assignments have a 20 year duration except for profile 3 which had a 40 year duration.

^2^ Air purifiers provided to diplomats for use in their residence while working in cities in Africa and Asia. Cities where air purifiers were provided are indicated with an asterisk (*).

**Supplementary Table 2: Example calculations of the impacted MR for COPD using inception and cessation lags**

Inception lag

In 2000, a person moved from Washington, DC to Manila, Philippines. In 1999, Washington, DC’s annual PM_2.5_ mean was 13.2 µg/m^3^ and the GBD CR function (reported as relative risk (RR)) of COPD mortality was 1.09. In 2000, Manila’s annual PM_2.5_ mean was 19.0 µg/m^3^ and the GBD CR function RR of COPD mortality was 1.15. To calculate the adjusted RR of COPD mortality in 2000, the 1^st^ year in Manila:

1. Lag the risk for Manila first by dividing the Manila RR by the Washington, DC RR and identify the excess risk due to Manila by subtracting 1
   - (1.15/1.09) - 1 = 0.05
2. Apply a 20-year inception lag to 0.05, the first year of lag is 30% of the lagged risk
   - (0.05 * 0.30) +1 = 1.02
3. Multiply the RR for Washington, DC (1.09) by the lagged risk for Manila RR/Washington, DC RR (this is 1.02 as indicated in step 2)
   - Adjusted RR in the first year in Manila = 1.09 * 1.02 = 1.11

5. Continue this calculation for 20 years each year up to 20 years in Manila or until the diplomat moves to an area with a change in PM_2.5_.

Cessation lag

In 2004, a person has lived in Riyadh, Saudi Arabia for 2 years and is moving to Munich, Germany. In 2003, Riyadh’s annual PM_2.5_ mean was 50 µg/m^3^ and the GBD CR function of COPD mortality for this PM_2.5_ level was 1.43. In 2004, Munich’s annual PM_2.5_ mean was 18.5 µg/m^3^ and the GBD CR function of COPD mortality for this PM_2.5_ level was 1.14. To calculate the adjusted RR of COPD mortality for 2004, the 1^st^ year in Munich:

1. Because the person had only lived in Riyadh for 2 years and had moved there from a city with much lower ambient PM_2.5_, the person’s evolved RR of COPD mortality was 1.25 and not the full RR for Riyadh, 1.43
2. Lag the continued risk for Riyadh by dividing the evolved Riyadh RR divided by the Munich RR and identify the excess risk due to Riyadh by subtracting 1
   - - (1.25/1.14) - 1 = 0.09
3. Apply a 20-year cessation lag to 0.09, the first year of the cessation lag is 70% of the lagged risk
   - (0.09 * 0.70) + 1 = 1.07
4. Multiply the RR for Munich (1.14) by the lagged risk for evolved Riyadh RR/Munich RR (1.07)
   - 1.14 * 1.07 = 1.22
5. Continue this calculation for the remaining 19 years, unless the person moves again

**Supplementary Table 3: Assumptions of the model and the sensitivity analysis**

- All diplomats move on 1 January each year and spend an entire year in a location.
- All diplomats live in the US before and after their 20 year period of diplomatic assignments and their PM_2.5_ exposure in the US is equal to the overall US annual mean concentration of PM_2.5_ in those years.
- An inception lag is applied to the GBD C-R function when a diplomat move to a location with higher ambient PM_2.5_ concentration compared to the prior location by 5 µg/m^3^ or more.
- A cessation lag is applied to the GBD C-R function when a diplomat move to a location with lower ambient PM_2.5_ concentration compared to the prior location by 5 µg/m^3^ or more.
- When a diplomat move to a location with a change in ambient PM_2.5_ concentration +/-5 ug/m^3^, the current lag is cancelled and a new lag is applied to take into account the difference between the RR in the new location and the evolved RR in the prior year in the prior location.
- Exposure to PM_2.5_ is estimated by city averaged annual PM_2.5_ concentration without considering the time spent outdoors or indoors, or activities including cooking or outdoor exercise that could potentially lead to a higher personal exposure to PM_2.5_.
- The GBD C-R functions apply to the US diplomatic population.
- Baseline mortality for the US diplomatic corps population is same as that for the US population, reflected in the US mortality statistics in 2019.
- The PM_2.5_ concentration and response relationship does not differ by sources of PM_2.5_.
- For child assignment profiles, PM_2.5_ exposure begins at birth excluding the pre-natal exposure period and continues until age 20 years in order to maintain the same duration of exposure among the three age groups studied.


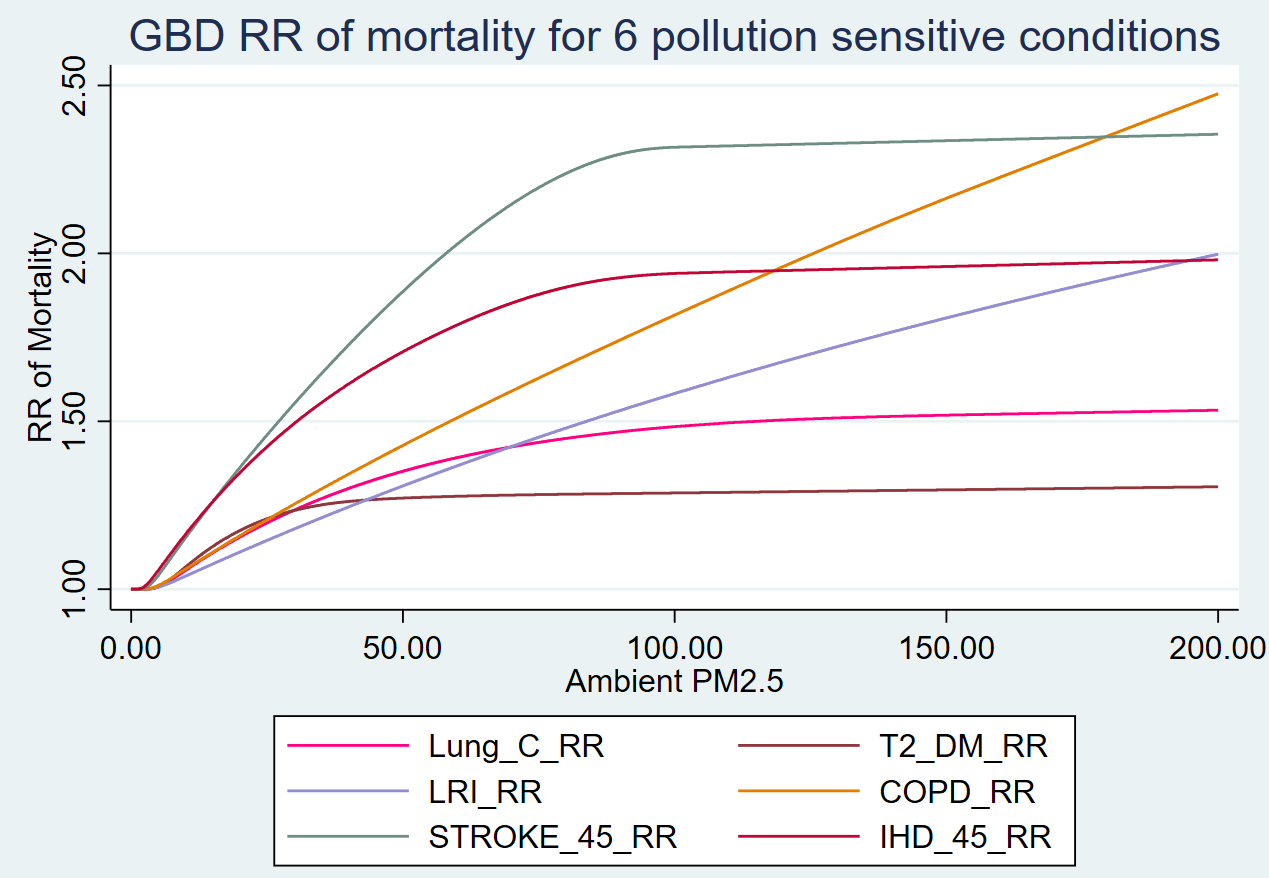


**Supplementary Figure 1: Global Burden of Disease 2019 concentration-response (C-R) functions for 6 pollution-sensitive conditions**

| **A – Inception Lags**  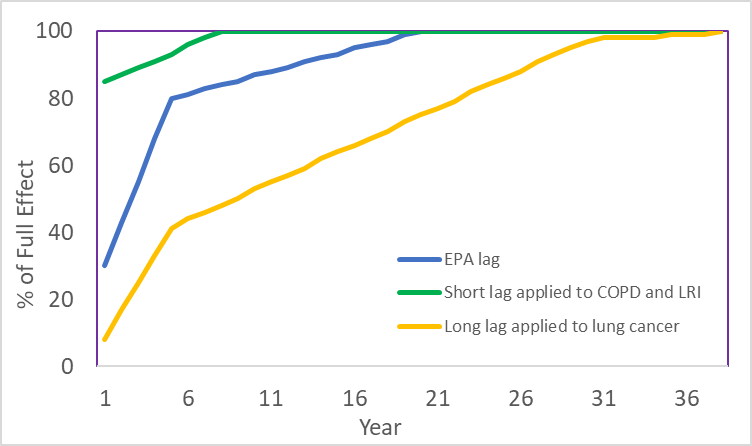 |
| --- |
| **B – Cessation Lags**  **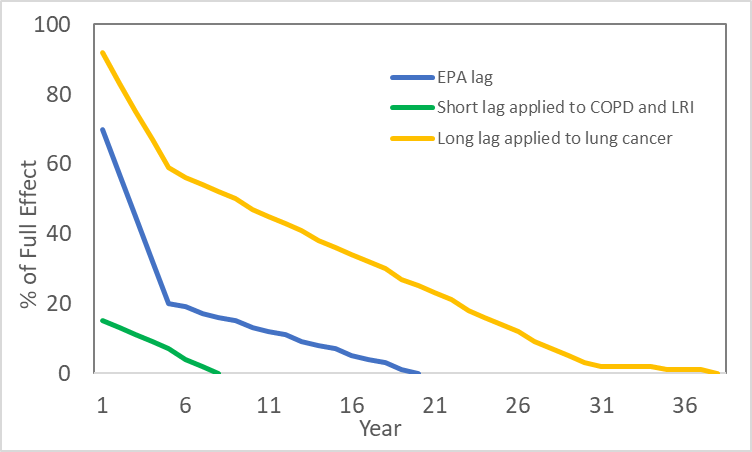** |

**Supplementary Figure 2**: **Inception (A) and cessation (B) lags used in the model and sensitivity analysis**

| (A) IHD  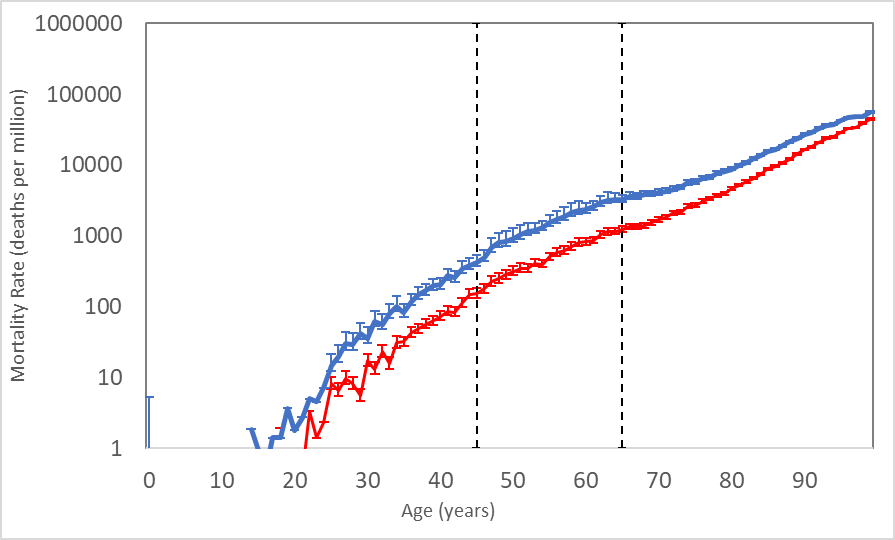 | (B) Stroke  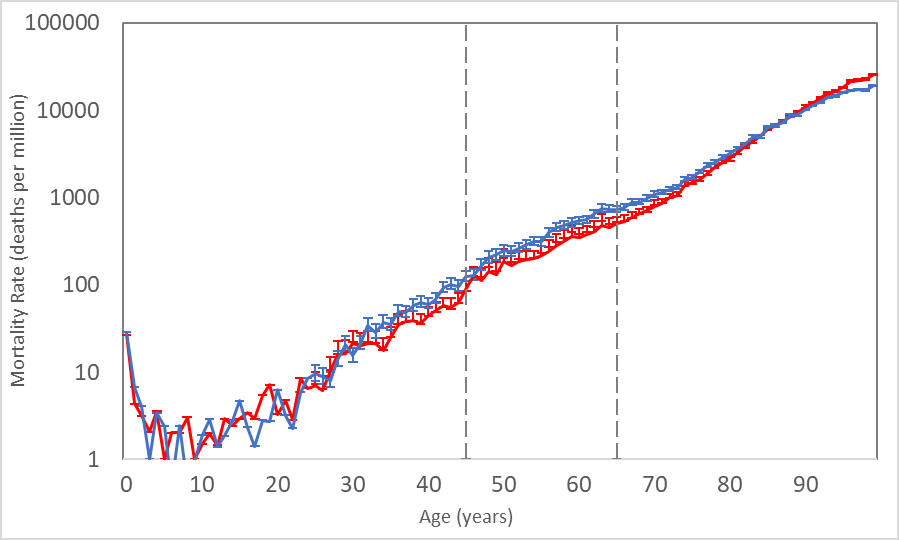 |
| --- | --- |
| (C) COPD  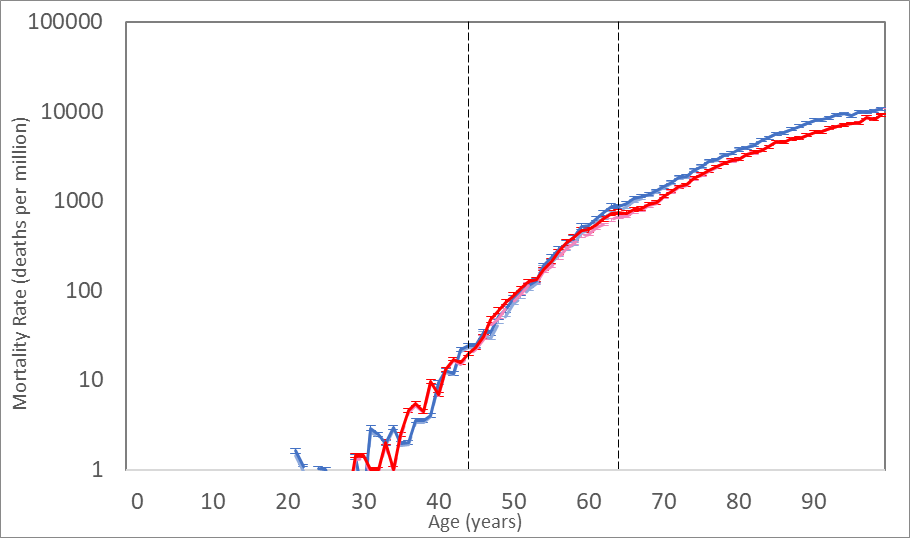 | (D) LRI  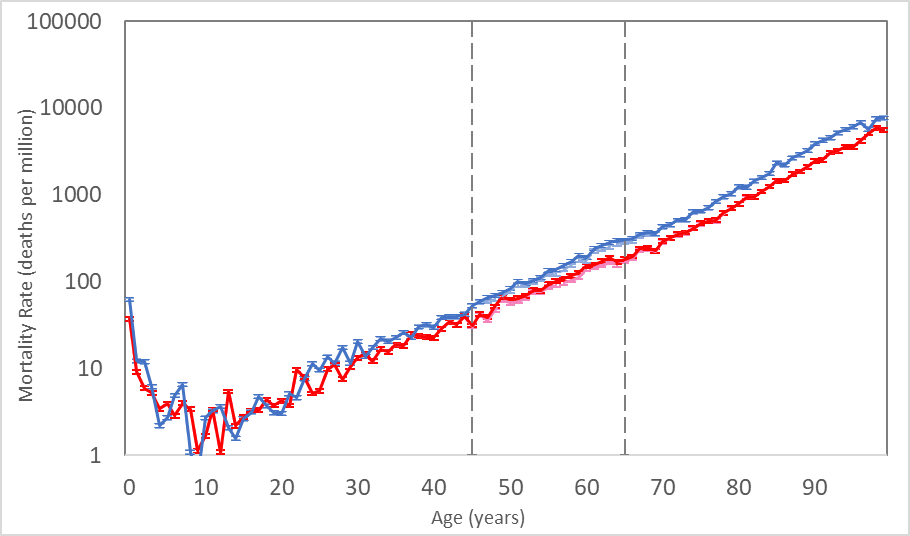 |
| (E) Type 2 Diabetes Mellitus  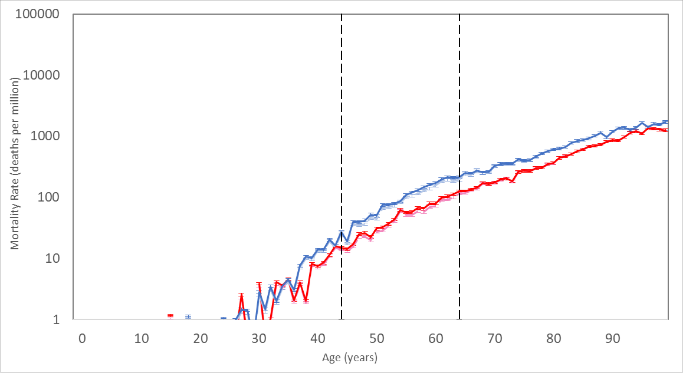 | (F) Lung Cancer  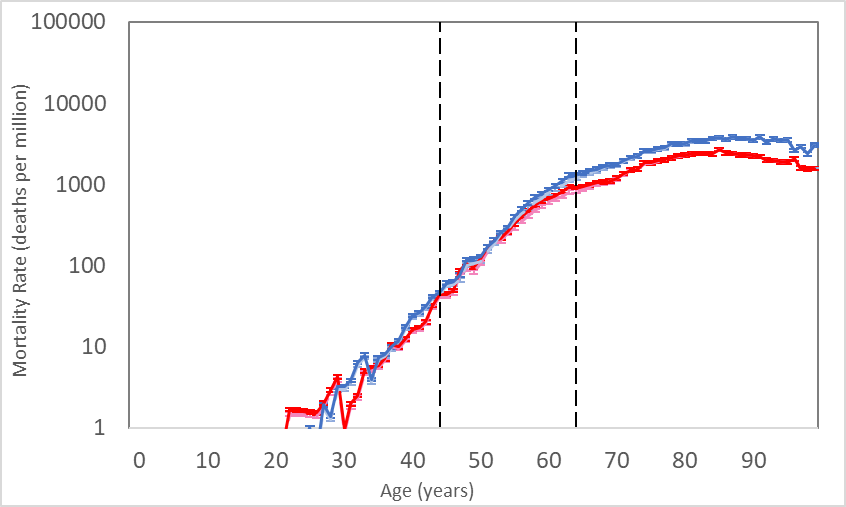 |
| 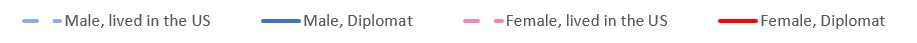 | |

**Supplementary Figure 3**: **Mortality rates for six pollution-sensitive conditions, older diplomat, Standard A assignment**
